# Role of COVID‐19 in the Progression of Scleroderma Interstitial Lung Disease and New Onset Pulmonary Hypertension: A Challenging Case Report

**DOI:** 10.1002/rcr2.70197

**Published:** 2025-05-06

**Authors:** Marco de Pinto, Amelia Spinella, Stefania Cerri, Martina Orlandi, Nicholas Landini, Elisabetta Olivi, Gabriele Amati, Ottavio Secchi, Giovanni Della Casa, Gilda Sandri, Enrico Clini, Clodoveo Ferri, Dilia Giuggioli

**Affiliations:** ^1^ Rheumatology Unit University Hospital of Modena and Reggio Emilia Modena Italy; ^2^ Respiratory Diseases Unit and Centre for Rare Lung Diseases University Hospital of Modena Modena Italy; ^3^ Department of Medical and Surgical Sciences for Children and Adults University of Modena and Reggio Emilia Modena Italy; ^4^ Department of Radiological Sciences, Oncology and Pathology Sapienza University of Rome Rome Italy; ^5^ Radiology Unit University of Modena and Reggio Emilia Modena Italy; ^6^ Rheumatology Clinic ‘Madonna Dello Scoglio’ Cotronei Crotone Italy

**Keywords:** COVID‐19, interstitial lung disease, pulmonary hypertension, Sars‐Cov2, systemic sclerosis

## Abstract

This case report describes an 80‐year‐old woman with systemic sclerosis (SSc), complicated by interstitial lung disease (ILD) and new‐onset pulmonary hypertension (PH), likely triggered by an atypical SARS‐CoV‐2 infection. Diagnosed with SSc in 2016 and previously stable ILD, she experienced clinical deterioration in 2023 with worsening respiratory failure and PH. Despite negative PCR and antigen tests, high anti‐SARS‐CoV‐2 IgG levels and CT findings were consistent with COVID‐19‐related organising pneumonia. Intravenous glucocorticoids led to partial symptom improvement, although ILD progression continued. The patient died in December 2023 from pneumococcal pneumonia. This case highlights the complex interaction between SSc and COVID‐19, where overlapping mechanisms of endothelial injury and fibrosis may exacerbate pre‐existing organ damage. It underscores the need for a multidisciplinary approach and timely interventions, including vaccination, early clinical assessment and appropriate immunosuppressive or antiviral treatments, to prevent severe infectious complications and halt disease progression in this high‐risk patient population.

## Graphical abstract

This report describes a woman with SSc and mild interstitial lung disease who developed COVID‐19 pneumonia, leading to rapid ILD progression and new onset pulmonary hypertension, ultimately resulting in fatal pneumococcal infection. The case underscores the critical role of multidisciplinary care and timely vaccination in managing high‐risk SSc patients during and after SARS‐CoV‐2 infection.

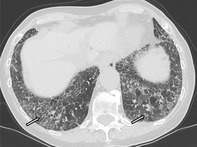

## 1 Introduction

Systemic sclerosis (SSc) is a chronic autoimmune disease marked by vasculopathy and fibrosis, with ILD occurring in about 50% of patients and contributing to high morbidity and mortality. The COVID‐19 pandemic has raised substantial concerns among rheumatologists about SSc patients due to the similar pathophysiological mechanisms shared by these conditions. Both SSc and COVID‐19 may be complicated by severe microvasculopathy and/or exacerbation or new onset ILD [1]. Notably, COVID‐19 has been shown to increase the risk of co‐infections, morbidity and mortality in SSc patients, particularly those with pre‐existing ILD [2]. This report describes a complex case of an SSc patient developing COVID‐19 pneumonia and consequent progression of ILD together with new onset pulmonary hypertension (PH).

## 2 Case Report

**FIGURE 1:**
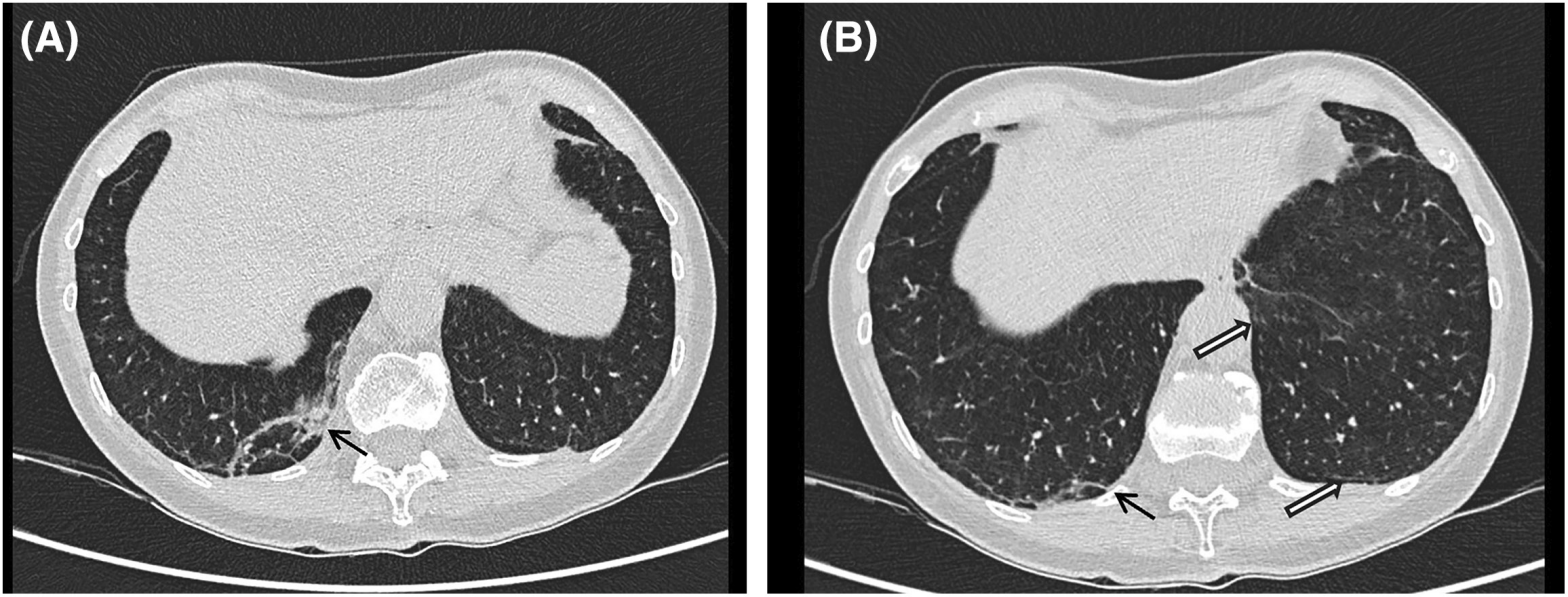
(a) Lung HRCT 2019: Tiny alterations, interpreted as mild ILD, are appreciable mainly at lung periphery of middle and lower zone (white arrows). (b) An atelectatic band is also present in the right lower lobe (black arrows).

The patient, an 80‐year‐old woman with a history of smoking, was diagnosed with SSc in 2016 due to the presence of Raynaud's phenomenon, puffy hands, an active SSc pattern on nailfold videocapillaroscopy and positive anti‐centromere antibodies. In 2019, thoracic high‐resolution computed tomography (HRCT) indicated mild ILD changes (Figure 1a,b). Initial treatment included calcium channel blockers and intravenous prostanoids, later discontinued due to side effects. The patient received four doses of the Pfizer‐BioNTech (BNT162b2) anti‐SARS‐Cov2 vaccine.

In 2021, she developed permanent atrial fibrillation unresponsive to cardioversion; aside from this, her condition remained stable.

**FIGURE 2:**
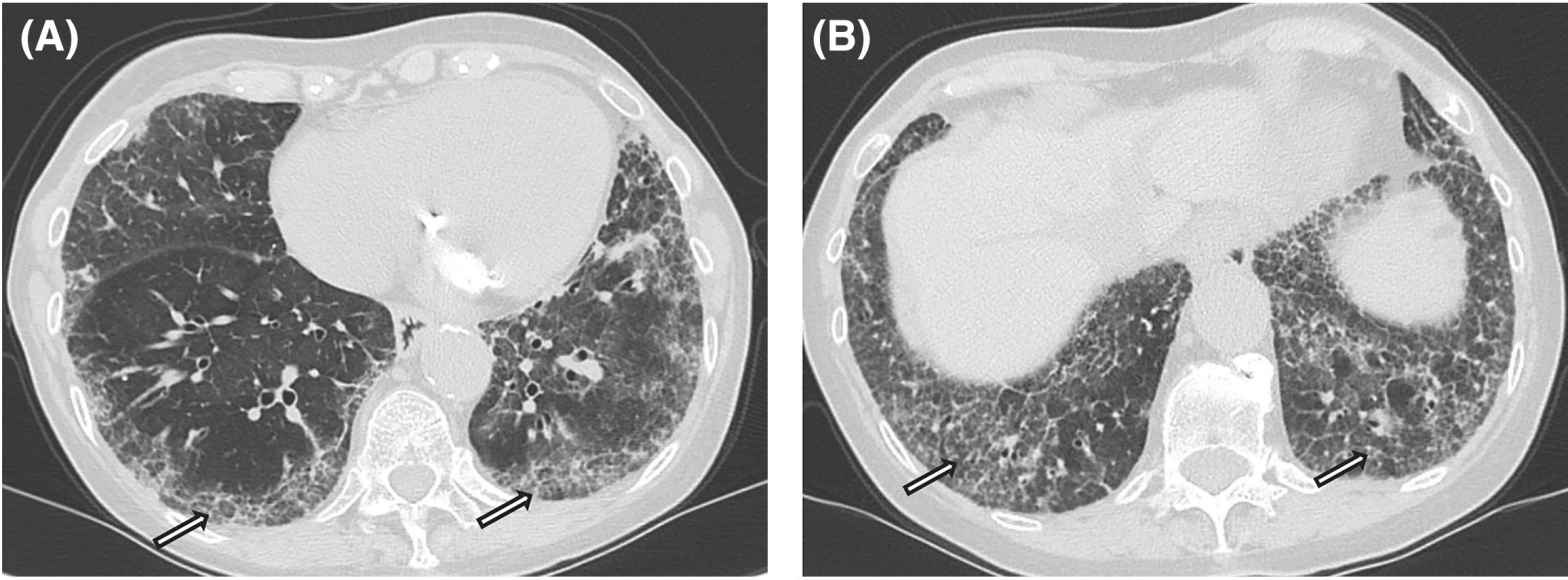
(a) Lung HRCT, April 2023, showed new lung alterations, sustained by ground glass opacities, reticulations and crazy paving appearance (white arrows). (b) Basal and peripheral distribution is predominant.

In April 2023, the patient was admitted to the emergency department with asthenia, high fever and respiratory failure. HRCT revealed significant lung abnormalities, including ground‐glass opacities, reticulations and crazy paving appearance (Figure 2a,b). Pulmonary function tests (PFTs) showed normal spirometry but severely reduced diffusing capacity for carbon monoxide (DLCO at 28%).

Testing for ongoing infections, including SARS‐CoV‐2 RNA detection by RT‐PCR on nasopharyngeal swab, was negative; after antibiotic treatment, she was discharged with home oxygen therapy.

**FIGURE 3:**
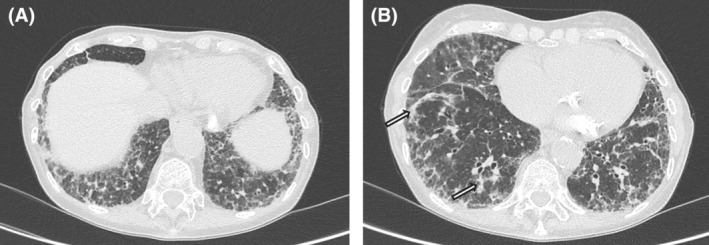
(a) Lung HRCT, July 2023: The extent of disease is increased. (b) Some perilobular bands of consolidations, resembling organising pneumonia, are appreciable (white arrows).

By July 2023, cardiac assessment, due to worsening of dyspnea, revealed moderate–severe mitral regurgitation, dilated and hypokinetic right ventricle, with a tricuspid regurgitation gradient suggesting elevated pulmonary pressures (ePAP at 75 mmHg). Right heart catheterisation confirmed combined pre‐ and post‐capillary PH (mPAP 31 mmHg, PVR 4.4 WU, WP 18 mmHg). Meanwhile, control lung HRCT showed progression of ILD (Figure 3a,b).

In August 2023, the patient was admitted to our Rheumatology Unit. The patient remained apyretic and presented with exertional dyspnea and a dry cough, with resting desaturation that required 2 L/min oxygen therapy. Apart from these symptoms, there were no additional clinical changes, such as the development of digital ulcers. PFTs revealed further deterioration in DLCO (17%) while spirometry remained normal. Given the patient's clinical condition, capillaroscopy was not repeated. Laboratory findings indicated elevated ESR and CRP, increased leukocyte count and high serum levels of anti‐SARS‐CoV‐2 IgG antibodies (anti‐IgG 28,000 BAU/mL). The patient continued to test positive for CENP‐B antibodies.

**FIGURE 4:**
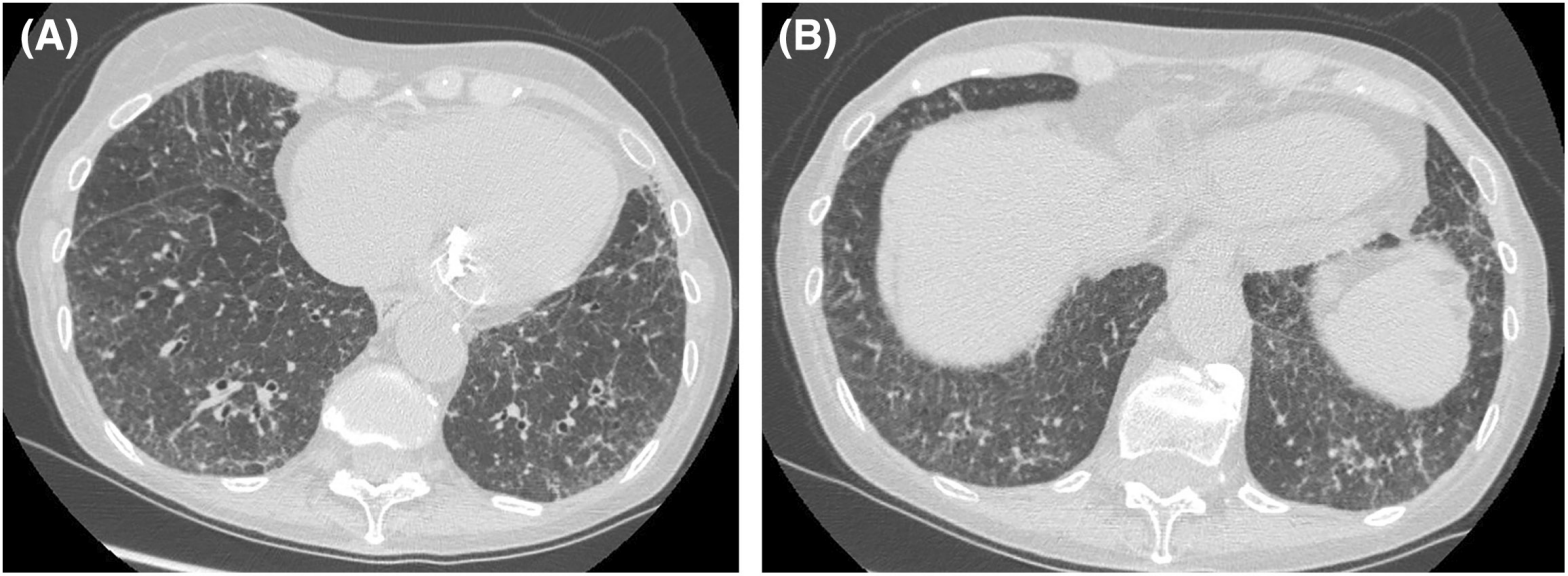
(a) Lung HRCT, November 2023: Reduction of lung abnormalities, still predominant at the periphery of lung bases. (b) Ground glass opacities are the main alterations.

A multidisciplinary team of rheumatologists, pulmonologists, cardiologists and radiologists reviewed the case. Based on clinical, laboratory and radiological findings, a diagnosis of organising pneumonia very likely due to COVID‐19 was made. The patient was treated with intravenous methylprednisolone (40 mg/day, gradually tapered to 5 mg/day), resulting in symptom improvement. Three months later, a follow‐up HRCT showed reduced ground‐glass opacities and resolution of parenchymal consolidation, though progression of ILD was confirmed (Figure 4a,b).

The team recommended pneumococcal and influenza vaccines before starting immunosuppressive therapy with mycophenolate mofetil; unfortunately, shortly after, the patient died from pneumococcal pneumonia before receiving the vaccine.

## 3 Discussion

The present report highlights the peculiar clinical history of a scleroderma patient with severe pneumonia attributable to COVID‐19 that resulted in a rapid progression of pre‐existing mild ILD and new‐onset PH.

The multidisciplinary approach allowed for consideration of the COVID‐19 responsible for the marked deterioration of the patient's cardiorespiratory conditions; this adverse evolution may be regarded as the consequence of virus‐driven vasculopathy and pneumonia overlapping with pre‐existing scleroderma tissue damage. At onset, CT helped rule out other conditions (e.g., pulmonary edema). However, COVID‐19 pneumonia can resemble SSc‐ILD on imaging, as both may show ground‐glass opacities with subpleural and basal distribution [3]. Disease progression or ILD exacerbation may further overlap, with features of organising pneumonia. Hence, a multidisciplinary approach is crucial.

In our case, the presenting acute inflammatory symptoms together with radiological findings of organising pneumonia and rapid response to steroid treatment supported the role of COVID‐19, confirmed by high serum levels of anti‐SARS‐CoV‐2 antibodies, in the rapid progression of pre‐existing scleroderma cardio‐respiratory manifestations.

The combination of ILD evolution and new onset PH further supports the hypothesis of the preeminent role of COVID‐19, in accordance with the well‐known ability of SARS‐CoV‐2 infection to trigger either lung profibrotic macrophage responses or endothelial cell injury with diffuse microangiopathy [1]. In our SSc patient, both pathogenetic mechanisms may have been responsible for acute respiratory distress syndrome as well as severe long‐term sequelae, such as cardiorespiratory failure, that lastly favoured the fatal pneumococcal infection. Such poor outcomes have not rarely been observed during the last pandemic in SSc patients [2]. Many adverse prognostic factors, namely a pre‐existing ILD, older age, smoking habits, diabetes, cardiovascular disorders and PH [4], were present in our patient.

COVID‐19 and SSc share similar physiopathological alterations, such as diffuse microvasculopathy and neofibrogenesis [2]; in this regard, SARS‐CoV‐2 infects both endothelial cells and macrophages causing diffuse endotheliitis with thrombotic manifestations, as well as a specific lung CD163+ macrophage polarisation driving excessive fibroproliferation [1].

Furthermore, SARS‐CoV‐2 infection per se may be responsible for an autoreactive immune response able to reactivate/amplify the SSc pathogenetic pathways.

Moreover, cases of SSc development after SARS‐CoV‐2 infection have been reported, suggesting an intriguing connection between these two conditions [5]. The worse outcome due to bacterial pneumonia emphasises the importance of pneumococcal vaccination, which is especially recommended for elderly people with important comorbidities who are particularly vulnerable to both viral and bacterial infections.

Finally, the case here described confirms the multifactorial/multistep pathogenesis of SSc for which novel intervening exogenous factors such as SARS‐CoV‐2 infection can negatively affect the natural course of the disease.

## Author Contributions

**Marco de Pinto:** conception and drafting of the work, acquisition, analysis and interpretation of data for the work. **Amelia Spinella:** conception and reviewing of the work, acquisition, analysis and interpretation of data for the work. **Stefania Cerri:** conception of the work, acquisition, analysis and interpretation of data for the work. **Martina Orlandi:** reviewing critically the work for important intellectual content. **Nicholas Landini:** analysis and interpretation of data and reviewing critically the work. **Elisabetta Olivi:** initial drafting of the work. **Gabriele Amati:** reviewing critically the work for important intellectual content. **Ottavio Secchi:** proofreading of the English text. **Giovanni Della Casa:** analysis and interpretation of data. **Gilda Sandri:** reviewing critically the work for important intellectual content. **Enrico Clini:** reviewing critically the work for important intellectual content and final approval of the version to be published. **Clodoveo Ferri:** reviewing critically the work for important intellectual content and final approval of the version to be published. **Dilia Giuggioli:** reviewing critically the work for important intellectual content and final approval of the version to be published.

## Consent

The authors declare that written informed consent was obtained for the publication of this manuscript and accompanying images and attest that the form used to obtain consent from the patient complies with the Journal requirements.

## Conflicts of Interest

The authors declare no conflicts of interest.

## Data Availability

Data sharing not applicable to this article as no datasets were generated or analysed during the current study.
